# Co-expression of IL-21-Enhanced NKG2D CAR-NK cell therapy for lung cancer

**DOI:** 10.1186/s12885-023-11806-1

**Published:** 2024-01-23

**Authors:** Yan Zhang, Cong Zhang, Minghong He, Weipeng Xing, Rui Hou, Haijin Zhang

**Affiliations:** 1 Department of Oncology, Shenyang 242 Hospital, 110034 Shenyang, China; 2 Department of Oncology, Hospital of Chengdu University of Traditional Chinese Medicine, 610072 Chengdu, China; 3 Department of Respiratory and Critical Care Medicine, Yidu Central Hospital of Weifang, 262500 Weifang, China; 4 Geneis Beijing Co., Ltd., 100102 Beijing, China

**Keywords:** NKG2D, CAR-NK, IL-21, Lung cancer, AKT

## Abstract

**Background:**

Adoptive cell therapy has achieved great success in treating hematological malignancies. However, the production of chimeric antigen receptor T (CAR-T) cell therapy still faces various difficulties. Natural killer (NK)-92 is a continuously expandable cell line and provides a promising alternative for patient’s own immune cells.

**Methods:**

We established CAR-NK cells by co-expressing natural killer group 2 member D (NKG2D) and IL-21, and evaluated the efficacy of NKG2D-IL-21 CAR-NK cells in treating lung cancer in vitro and in vivo.

**Results:**

Our data suggested that the expression of IL-21 effectively increased the cytotoxicity of NKG2D CAR-NK cells against lung cancer cells in a dose-dependent manner and suppressed tumor growth in vitro and in vivo. In addition, the proliferation of NKG2D-IL-21 CAR-NK cells were enhanced while the apoptosis and exhaustion of these cells were suppressed. Mechanistically, IL-21-mediated NKG2D CAR-NK cells function by activating AKT signaling pathway.

**Conclusion:**

Our findings provide a novel option for treating lung cancer using NKG2D-IL-21 CAR-NK cell therapy.

**Supplementary Information:**

The online version contains supplementary material available at 10.1186/s12885-023-11806-1.

## Introduction

Lung cancer is a common malignant tumor with high morbidity and mortality, which has attracted wide attention of researchers [1–4]. More and more treatments have been applied to lung cancer, such as surgery, radiation therapy, antibody therapy, and chemotherapy [5–9]. However, the survival rate of patients with lung cancer remains very low, and the five-year survival of these pates less than 25.0% [10–13]. Hence, novel treatment of lung cancer is urgently needed.

Recently, adoptive cell therapy (ACT) has been used to treat B-cell acute lymphoblastic leukemia (B-ALL) by genetically engineering autologous T cells expressing chimeric antigen receptors (CAR) with specificity for *CD19*, and showed remarkable curative effects [14, 15]. However, CAR-T cell therapy is limited to self-treatment because allogeneic CAR-T cells from donors may be rapidly eliminated by the host immune system, cause life-threatening graft-versus-host disease (GvHD) and cytokine storm [16, 17].

Natural killer (NK) cells are innate lymphocytes that have been used in many clinical trials due to they are endowed with potent cytotoxic activity including the direct release of perforin and granzymes, carrying out antibody-dependent cellular cytotoxicity (ADCC) through the CD16 membrane receptor, and utilizing Fas ligand (FasL) or TNF-related apoptosis-inducing ligand (TRAIL) to trigger target cell apoptotic pathways [18], which could target cancer cells in an antigen-independent and non-major histocompatibility complex (MHC)- restricted manner [19]. Clinically, allogeneic NK cells (NK cell line NK-92) have been broadly used for CAR-NK cell engineering for not causing GvHD [20, 21]. Besides, NK cells have a better safety profile than T cells because NK cells can lack clonal expansionand reduce cytokine storm [22]. To obtain a sufficient number of NK cells for clinical therapy, various approaches are employed, including culturing NK cells in cytokine mixtures comprising IL-2, IL-12, IL-15, IL-18, and/or IL-21, or utilizing K562 cell-based cytokine-transfected cells (e.g., incorporating IL-15 or IL-21 along with 4-1BBL) [23, 24]. Furthermore, advancements in gene modification technologies have enabled the generation of CAR-NK cells through viral transfection methods such as lenti- or retroviruses, as well as non-viral approaches involving naked plasmid DNA, transposase DNA-mediated integration, or mRNA transfection [22]. Despite these developments, the immunosuppressive tumor microenvironment (TME) often poses challenges to NK cell responsiveness. Upon infiltration into the TME, the phenotype and metabolism of NK cells undergo alterations, leading to impaired cytotoxicity and reduced expression of activating receptors, including DNAM1, NKp80, NKp30, and CD16. Additionally, there is an upregulation in the expression of molecules associated with NK cell exhaustion, such as PD-1, CD96, Tim3, and TIGIT [25, 26]. In addition, Moreover, CAR-NK cell therapy targeting ROBO1, ErbB2/HER2, EGFR, EpCAM, and MUC1 are currently undergoing clinical studies in many cancer types [27–32]. Therefore, CAR-NK cells have a beneficial clinical application prospect in cancer treatment.

To improve the efficacy of CAR-NK cell therapy, an increasing number of therapeutic targets have been discovered. Natural killer group 2 member D (*NKG2D*), which is expressed by NK cells, CD8^+^ T cells, γδT cells, and NKT cells, is an activation receptor and can interact with eight NKG2D ligands (NKG2DLs), including MICA/B and UL16-binding proteins (ULBP1-6), which have been reported to be expressed on a variety of solid tumors and hematologic malignancies [33, 34]. *NKG2DLs* are ideal therapeutic target for anti-cancer strategies because they can be up-regulated upon infection, DNA damage, and transformation of cells [35, 36].

In order to increase the cytotoxicity of CAR-NK cells, many modifications can be added to the CAR structure [37–39]. For example, Interleukin-21 (IL-21), a pleiotropic cytokine, is primarily secreted by activated CD4^+^ T cells. IL-21 is involved in multiple biological processes via activating the JAK/STAT, MAPK and PI3K/Akt pathways, such as viral infections, autoimmune disorders, allergies, and cancer [40, 41]. The IL-21 receptor (IL-21R), is located on the surface of immune cells, and mediate a variety of biological functions, such as promoting the proliferation and cytotoxicity in CD8^+^ T cells, amplifying macrophage activation pathways, and increasing the expression of NK activation receptors in human NK cells [42–46].

In this study, we constructed NK-92 cells expressing NKG2D and IL-21 to investigate if efficacy against lung cancer. The anti-tumor efficacy of NKG2D-IL-21 CAR-NK cells has been evaluated compared with NKG2D CAR-NK-92 cells in vitro and in vivo. Our data demonstrated that NKG2D-IL-21 CAR-NK-92 cells have a promising anti-tumor activity for lung cancer, hence it will be a potential novel therapy for lung cancer.

## Materials and methods

### Cell cultures

NK-92 cells (directly purchased from ATCC) were maintained in α-MEM (GIBCO) supplemented with 12.5% FBS (GIBCO), 12.5% horse serum, IL-2 (100IU/ml) and 1% penicillin-streptomycin (GIBCO). A549, H1975 and PC9 cells were grown in DMEM (GIBCO) supplemented with 10% FBS and 1% penicillin-streptomycin. All cell lines were incubated at 37 °C in 5% CO_2_ and the medium was replaced every 2 days.

### Vector design

To generate the NKG2D CARs, the extracellular domain of human- synthesized NKG2D (Idobio, China) and cloned into a CAR-encoding lentivirus backbone, containing a CD8α hinge spacer and transmembrane domain, 4-1BB, and CD3ζ endo-domains. The full-length IL-21 vector, synthesized by Integrated DNA Technology, was subcloned into downstream of the NKG2D, which was linked with a modified 2 A peptide to generate NKG2D-IL-21CAR.

### Lentiviral package

The lentiviral plasmids and the packaging plasmids (psPAX2 and pMD2.G) were co-transfected into HEK293T cells at a ratio of 10:10:6. The transfected cells were incubated at 37 °C for 6–8 h, and then the fresh medium was added to the wells for another 48 h. Supernatants were collected, filtered through a 45 μm filter, and concentrated by ultracentrifugation at 15,000 rpm for 2.5 h at 4 °C. The viruses were aliquoted and stored at -80 °C.

### CAR-NK cells preparation

NK-92 cells were suspended in α-MEM and seeded into 12-well plates (0.5 mL/well, 4 × 10^5^cells), and then lentiviral particles were added to the cultures at a multiplicity of infection (MOI) of 10 in the presence of polybrene at a final concentration of 8 µg/mL, followed by centrifugation at 1800 × *g* for 1 h. After 1 d, the recombinant viruses were removed and CAR-NK cells were cultured to expand. The cells were collected and used for the in vitro experiments.

### Flow cytometry

Cell surface and intracytoplasmic marker expression were detected using a BD CantoII flow cytometer. For extracellular staining, anti-human CD56-FITC (eBiosciences, MA1-19129), NKG2D-PE (BD, 561,815), MICA/B-PE (Biolegend, 320,906), ULBP1-FITC (Invitrogen, MA5-38655), CD107a-APC (BD, 641,581), and TIM-3-PE-cy7 (Biolegend, 345,013) were used for extracellular staining. For intracellular staining, CAR-NK cells were fixed and permeabilized using a BD Cytofix/Cytoperm kit (BD Biosciences, Franklin Lakes, USA). following the manufacturer’s protocol, and anti-human IFN-γ-eFluor 450 (eBiosciences, 85-48-7319-42) was used for intracellular staining. Stained samples were acquired on a BD CantoII flow cytometry and analyzed with FlowJo software (Tree Star, Ashland, USA).

### Cytotoxicity assay

The anti-tumor activity of CAR-NK-92 cells was evaluated using flow cytometry. Firstly, A549, H1975 and PC9 cells (target cells) were stained with carboxyfluorescein succinimidyl ester (CFSE) and then seeded into low adsorption 96-well plates at a density of 2 × 10^4^ cells/well. Secondly, non-transduced NK-92 cells or NKG2D-BBz CAR-NK cells (effector cells) were added to each well to ensure an effector:target cell (E:T) ratio of 1:1, 2.5:1, or 5:1. After 4 h of co-culture, the tumor cells and effector cells were collected, and dead cells were stained with Annexin V FIFC (BD-Pharmingen, 556,547) and PI.

### Proliferation assay

Mock NK-92, NKG2D CAR-NK-92, and NKG2D-IL-21 CAR-NK-92 cells were labeled with CFSE and co-incubated with A549 at a 1:10 ratio for 6 d. The cell counts and CFSE dilution was measured by BD CantoII flow cytometry.

### Western blotting

The RIPA lysate was used to extract the total protein from Mock NK-92, NKG2D CAR-NK-92, and NKG2D-IL-21 CAR-NK-92 cells and then centrifugated at 12,000 rpm at 4℃for 10 min. The supernatants were subjected to 12.5% sodium dodecyl sulfate polyacrylamide gel electrophoresis (SDS-PAGE) and transferred to polyvinylidene difluoridefilter (PVDF) membranes. After blocking with 5% dried skim milk, the membranes were incubated with IL-21 mAb primary antibodies and actin overnight at 4 °C. Subsequently, the membranes were incubated with the secondary antibody for 1 h at room temperature. The density of the bands was quantified by normalization to GAPDH using Image J Software (National Institutes of Health, Bethesda, MD, USA).

### Enzyme-linked immunosorbent assay

Mock NK-92, NKG2D CAR-NK-92, and NKG2D-IL-21 CAR-NK-92 cells were prepared and incubated for 12 h. The cytokine of IL-21 or IFN-γ concentration in the medium was measured using the enzyme-linked immunosorbent assay (ELISA) kits (Pierce Endogen, Rockford, IL) according to the manufacturer’s instructions.

### Xenograft mouse model

Eight-week-old female NSG (NOD-PrkdcscidIL2rgtm1/Bcgen, Beijing Biocytogen Co., Ltd, China) mice were used to assess the CAR-NK-92 cells in vivo. The mice were randomly divided into three groups (*n* = 5 each group) and inoculated subcutaneously (s.c.) into the right flank with 4 × 10^6^ A549 cells in 100 µL of 50:50 Matrigel (Corning) and phosphate-buffered saline (PBS). One week after infusion, mice were intravenously injected with 10^7^ NKG2D CAR-NK-92 cells. Eight-week-old female C57bl/6, Beijing Biocytogen Co., Ltd, China) mice were used to assess the mCAR-NK cells in vivo. The mice were randomly divided into three groups (*n* = 5 each group) and inoculated subcutaneously (s.c.) into the right flank with 5 × 10^6^ Lewis cells in 100 µL of 50:50 Matrigel (Corning) and phosphate-buffered saline (PBS). One week after infusion, mice were intravenously injected with 10^7^ mCAR-NK cells. Tumor dimensions were measured with the calipers, and the formula V = 1/2(length × width^2^) was used to calculate tumor volume. The production of IFN-γ in serum was detected using ELISA. All animal studies were carried out under protocols approved by the Institutional Animal Care and Use Committee of The First Affiliated Hospital of Soochow University.

### Statistical analysis

All data analyses were performed using Prism software 6.0 (GraphPad) and expressed as mean ± SD. Data were compared using the one-way ANOVA test. *p* < 0.05 indicated statistically significant difference. Comparison of survival curves was done using the log-rank test. Each experiment consisted of at least three replicates per condition.

## Results

### IL-21 is closely related to the infiltration of immune cells in lung cancer

**Fig. 1:**
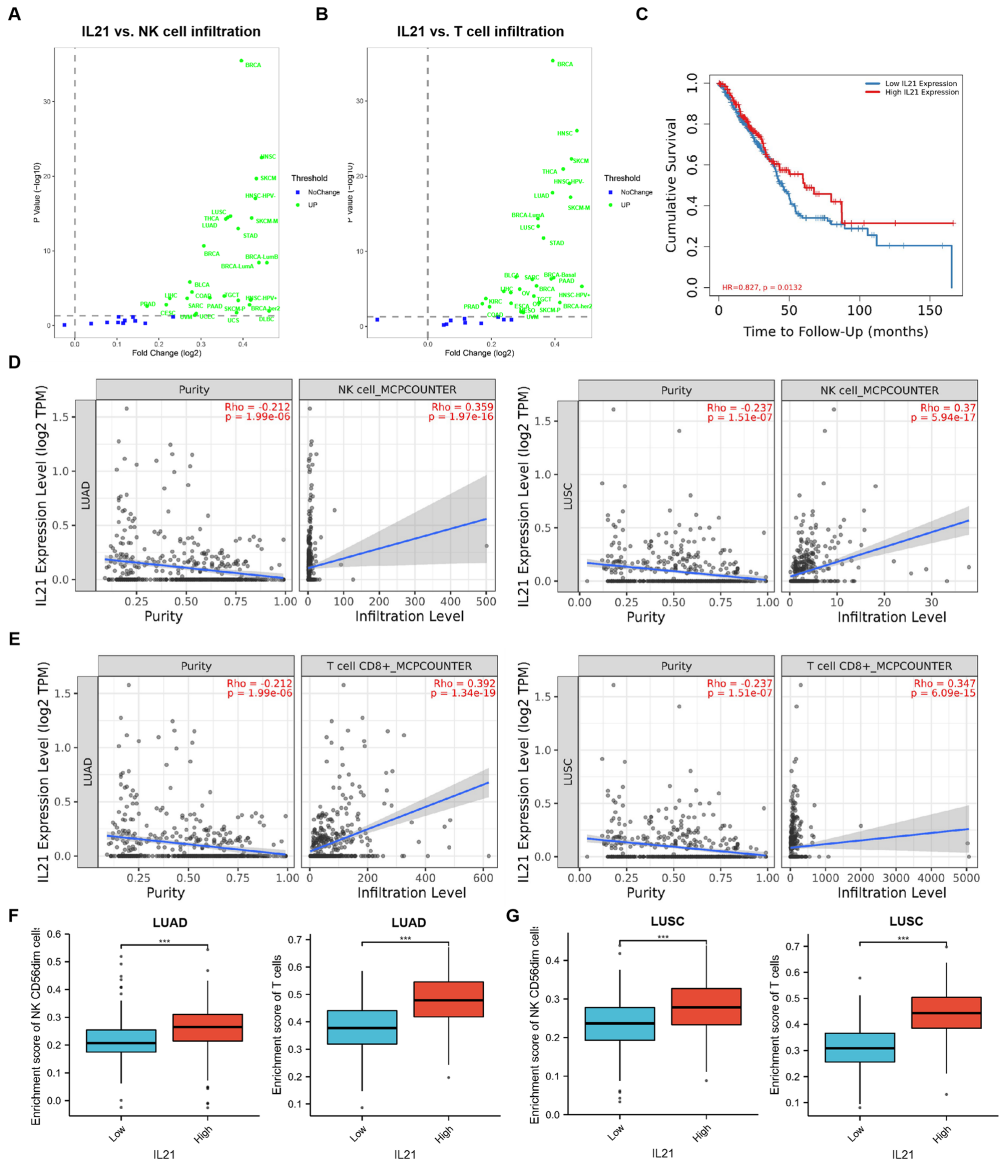
The relationship between the expression of IL-21 and the infiltrations of immune cells. (**A, B**) Scatter plots of the relationship between NK and T cells infiltrations and the IL-21 expression levels in cancers. (**C**) Kaplan-Meier survival analysis demonstrated that high IL-21expression levels notably correlated with good overall survival of patients with lung cancer. (**D, E**) Scatter plots of the relationship of NK and T cells infiltrations and the IL-21 expression levels in LUAD (**D**) and LUSC (**E**) seprately. (**F, G**) The relationship between high and low expression of IL-21 and the level of immune cell infiltration using the Tumor Immune Estimation Resource (TIMER2.0) web tool

We first used TIMER2 (tumor immune estimation resource, version 2) web (http://timer.cistrome.org/) to explore the relationship between the expression of IL-21 and the infiltration of immune cell in cancers. The data showed that IL-21 expression was positively correlated with the infiltrations of NK cells and T cells in a number of cancers (Fig. 1A, B). In addition, the higher the expression of IL-21 is, the higher the survival rate of lung cancer patients (Fig. 1C). The expression patterns of genes within the microenvironment can have different implications for tumor purity. Genes that are highly expressed in the microenvironment are anticipated to show negative associations with tumor purity, whereas genes highly expressed in the tumor cells are expected to have a positive correlation. In this context, we observed a positive correlation between the expression of IL-21 and the infiltrations of NK cells and T cells in both lung adenocarcinoma (LUAD) and lung squamous cell carcinoma (LUSC) (Fig. 1D, E). Furthermore, it was observed that higher expression levels of IL-21 were associated with increased infiltration of NK cells and T cells (Fig. 1F, G). These results suggested that IL-21 may play an important role in increasing the infiltration of immune cells in number of cancers.

### NKG2DL expression and structure of NKG2D-IL-21 CAR

**Fig. 2:**
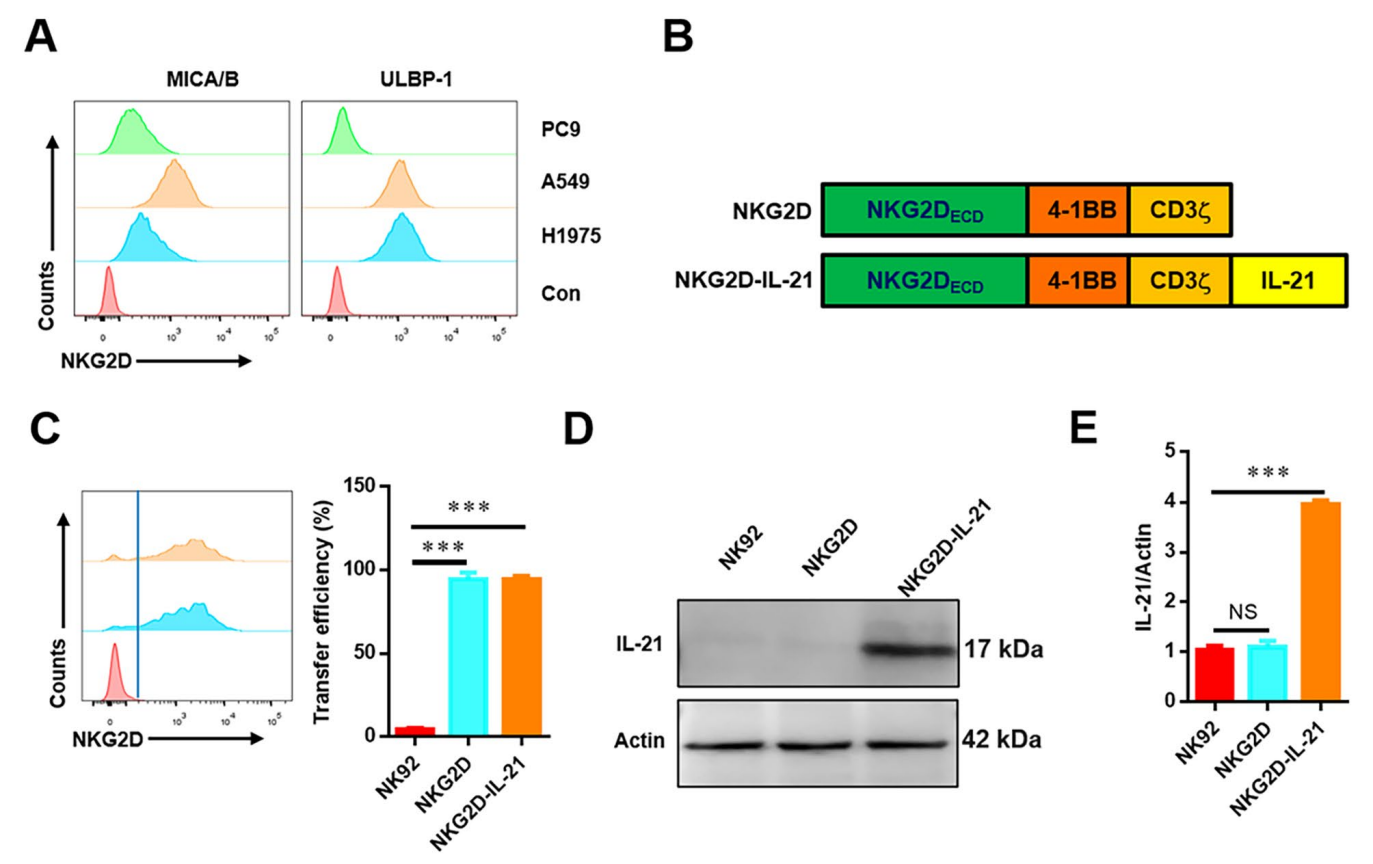
Generation and NKG2DLs expression of CAR-NK-92 cells. (**A**) NKG2DLs expressions in three lung cancer cell lines were quantified by flow cytometry. (**B**) Schematic representation of NKG2D. (**C**) Flow cytometry analysis of the transfection efficiencies. (**D**) Western blot analysis of IL-21 protein expression level in NK-92, NKG2D CAR-NK-92, and NKG2D-IL-21 CAR-NK-92 cells. **€** ELISA analysis of IL-21 protein expression level in NK-92, NKG2D CAR-NK-92, and NKG2D-IL-21 CAR-NK-92 cells. Data were representative for three independent experiments. *** *P* < 0.001; ns, not significant

Next, we adopted flow cytometry to assess cell surface expression of NKG2DLs, MICA/B and ULBP1 on the lung cancer cell lines including A549, H1975, and PC9. The results showed that the expression level of NKG2DLs is relatively high in most lung cancer cell lines. A549 and H1975 cells highly expressed MICA/B and ULBP1, while low levels of MICA/B and ULBP1 were detected on PC9 cells. Therefore, PC9 cells were used as a negative control in the subsequent experiments (Fig. 2A).

The NKG2D-IL-21CAR was constructed consisting of the extracellular domain (ED) of NKG2D, the CD8α hinge and transmembrane region, and followed by a 4-1BB intracellular signaling domain, the CD3ζ signaling moiety and *IL-21* (Fig. 2B). We generated human NKG2D CAR- or mock-transduced NK-92 cells by lentiviral transfection. Allophycocyanin (APC)-conjugated NKG2D antibody (clone 1D11, BioLegend) was used to measure the *NKG2D* transfection.As shown in Fig. 2C, the expression levels of NKG2D in NKG2D CAR-NK-92 cells were significantly higher than in mock-transduced NK-92 cells, indicating that NKG2D-based CAR was successfully transduced in NK-92 cells.

Since the overexpression of IL-21 in NKG2D CAR-NK-92 cells was an important factor in this study, we used western blotting (WB) and ELISA to determine the protein expression level of IL-21. Figure 2D**&E** and Supplementary Fig. 1 show that the expression levels of IL-21 in NKG2D-IL-21CAR-NK cells were significantly higher than in mock-transduced NK-92 cells and NKG2D CAR-NK-92 cells, indicating that NKG2D-IL-21 CAR-NK-92 cells were successfully established.

### NKG2D-IL-21 CAR-NK-92 cells effectively recognize and eliminate lung cancer cell lines in vitro

**Fig. 3:**
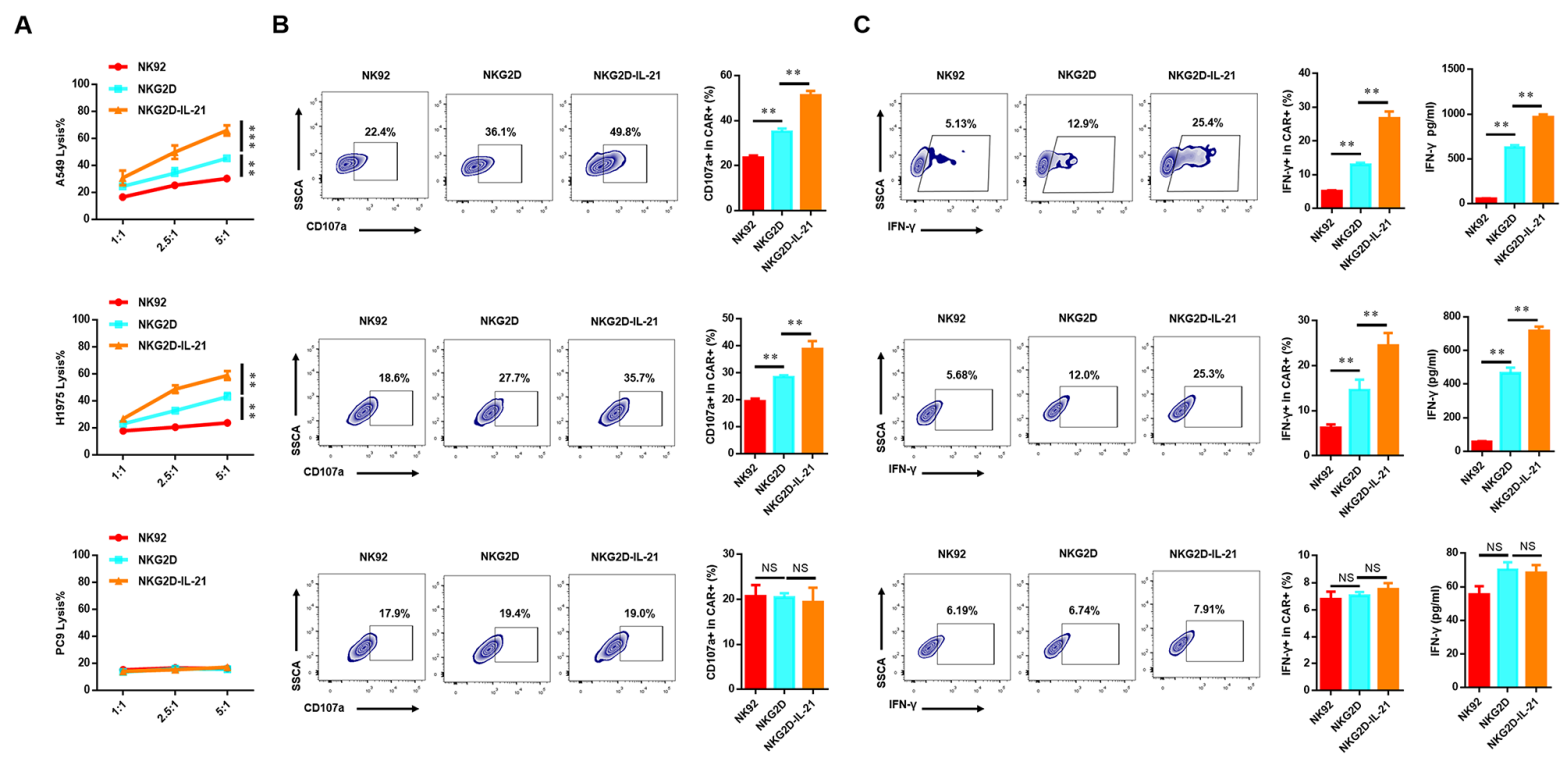
Estimation of cytotoxic activity in CAR-NK-92 cells. (**A**) Line plots showing the cytotoxicity of NK-92, NKG2D CAR-NK-92 cells, and NKG2D-IL-21 CAR-NK-92 cells against the lung cancer cell lines at a different effector to target (E: T) ratios for 4 h. The expression level of CD107a (**B**) and the released amount of IFN-γ (**C**) in CAR-NK-92 cells were measured after co-culturing with lung cancer cell lines at a 5:1 ratio for 4 h by flow cytometry. Data were representative of three independent experiments. ** *P* < 0.01; ns, not significant

Given the expression of IL-21 in NKG2D-IL-21 CAR-NK-92 cells and its established role in enhancing cytotoxic activity, our investigation focused on evaluating the in vitro anti-tumor effects of NKG2D-IL-21 CAR-NK-92 cells. To accomplish this, we conducted co-culture experiments by incubating genetically engineered NK-92 cells with various lung cancer cell lines in cytotoxicity assays. Compared to mock-transduced NK-92 cells, NKG2D CAR-NK92 cells displayed significantly higher cytolytic activity, while NKG2D-IL-21 CAR-NK-92 cells showed the highest cytolytic activity against NKG2DL (+) lung cancer cells, but there were not significant differences when co-cultured with the NKG2DL (−) lung cancer cells (Fig. 3A).

CD107a, a sensitive marker for the identification of the cytolytic activity of immune cells, is expressed temporarily on the cell surface when NK-92 cells produce lytic granules including granzymes and perforin to kill target cells (37). As shown in Fig. 3B, NKG2D-IL-21 CAR-NK-92 cells displayed significantly enhanced surface CD107a expression in response to co-culturing NKG2DL (+) lung cancer cells.

As an important effector molecule of CAR-NK cells, it has been reported that *IL-21* promotes the production of interferon-γ (IFN-γ) in NK-92 cells (38, 39). We evaluated if the NKG2D CAR-NK-92 cells produced an increased amount of IFN-γ. As shown in Fig. 3C, after co-cultured with NKG2DL (+) lung cancer cells, the expression level of IFN-γ in NKG2D-IL-21 CAR-NK cells was higher than that in NKG2D CAR-NK cells.

### NKG2D-IL-21 CAR-NK cells are more proliferative than NKG2D CAR-NK cells

**Fig. 4:**
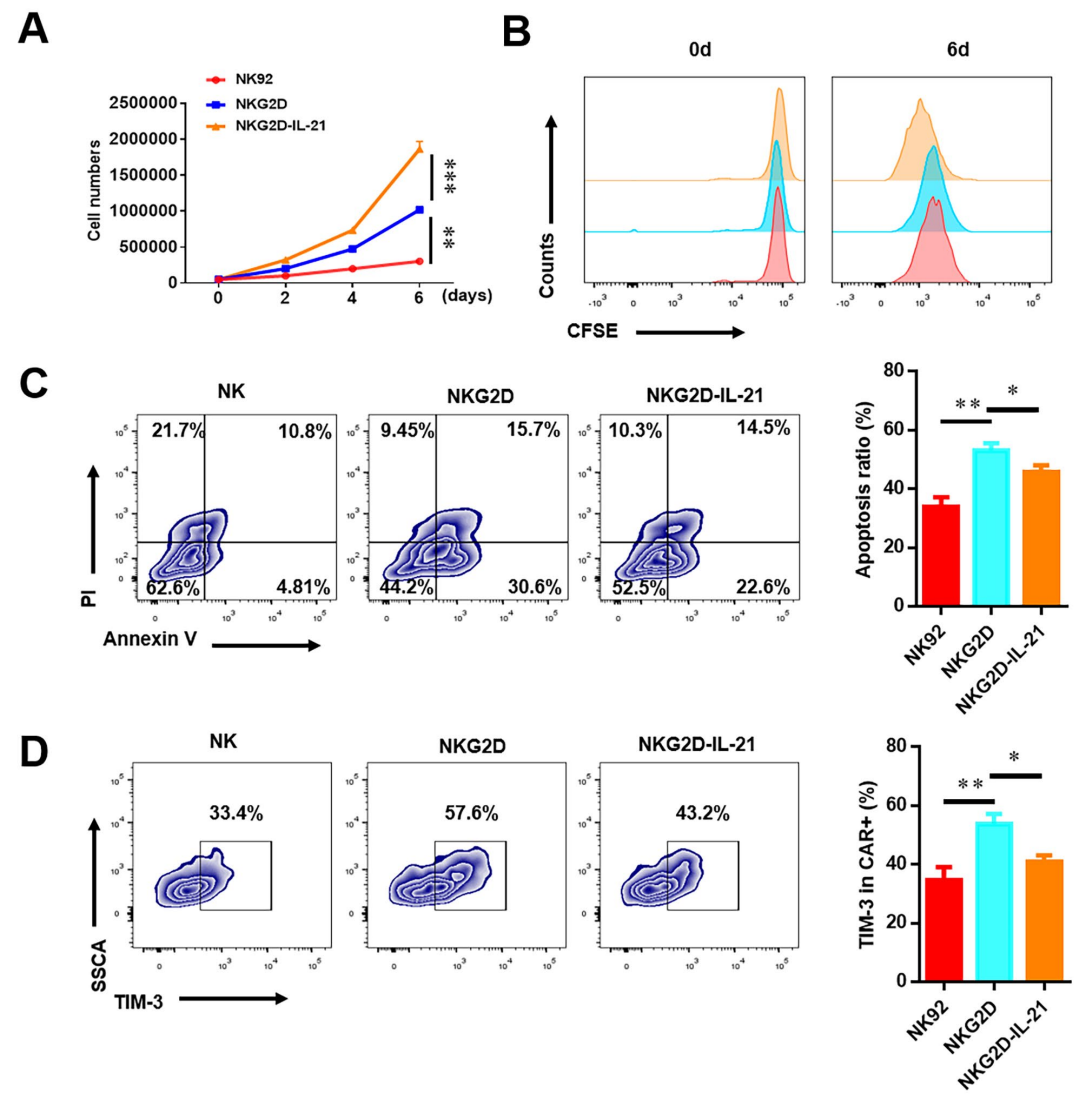
IL-21 enhanced proliferation potency and reduced autologous apoptosis and exhaustion of CAR-NK cells. (**A, B**) NK-92 cells, NKG2D CAR-NK-92 cells, and NKG2D-IL-21 CAR-NK-92 cells labeled with *CFSE*, were co-incubated with A549 cells at a 1:10 ratio for 6 d. The cell counts and CFSE dilution were measured. (**C, D**) NK-92, NKG2D CAR-NK-92, and NKG2D-IL-21 CAR-NK-92 cells which were co-incubated with A549 at a 1:10 ratio for 6 d, the apoptosis ratio and TIM-3 expression of CAR-NK-92 cells was detected by flow cytometry. Data were representative of three independent experiments. * *P* < 0.05; ** *P* < 0.01

IL-21 has been identified as a key regulator capable of augmenting proliferation and cytotoxicity in both NK cells and CD8 + T cells. As the proliferation of CAR-NK cells constitutes a critical determinant in anti-tumor immunity, this aspect gains particular significance. Accordingly, we observed that NKG2D-IL-21 CAR-NK-92 cells showed improved proliferation efficiency than NKG2D CAR-NK-92 cells after co-culturing with lung cancer cells (Fig. 4A**&B**). Apoptosis and exhaustion of CAR-NK-92 cells are important mechanisms for suppressing the anti-tumor immune response. T-cell immunoglobulin and mucin-domain containing-3 (TIM-3) is an inhibitory receptor expressed on NK cells, and could suppress NK cell-mediated anti-tumor immunity and reduce the expression of inhibitory receptors that contribute to enhancing the cytotoxic activity of NK-92 cells. To characterize NKG2D-IL-21 CAR-NK-92 cells apoptosis and exhaustion, the expression levels of apoptosis marker and TIM-3 were measured by flow cytometry. As shown in Fig. 4C**&D**, the expression levels of apoptosis markers and TIM-3 on NKG2D-IL-21 CAR-NK cells were lower than those in NKG2D CAR-NK-92 cells.

### IL-21 mediates CAR NK-92 cells via *AKT* signaling pathway

**Fig. 5:**
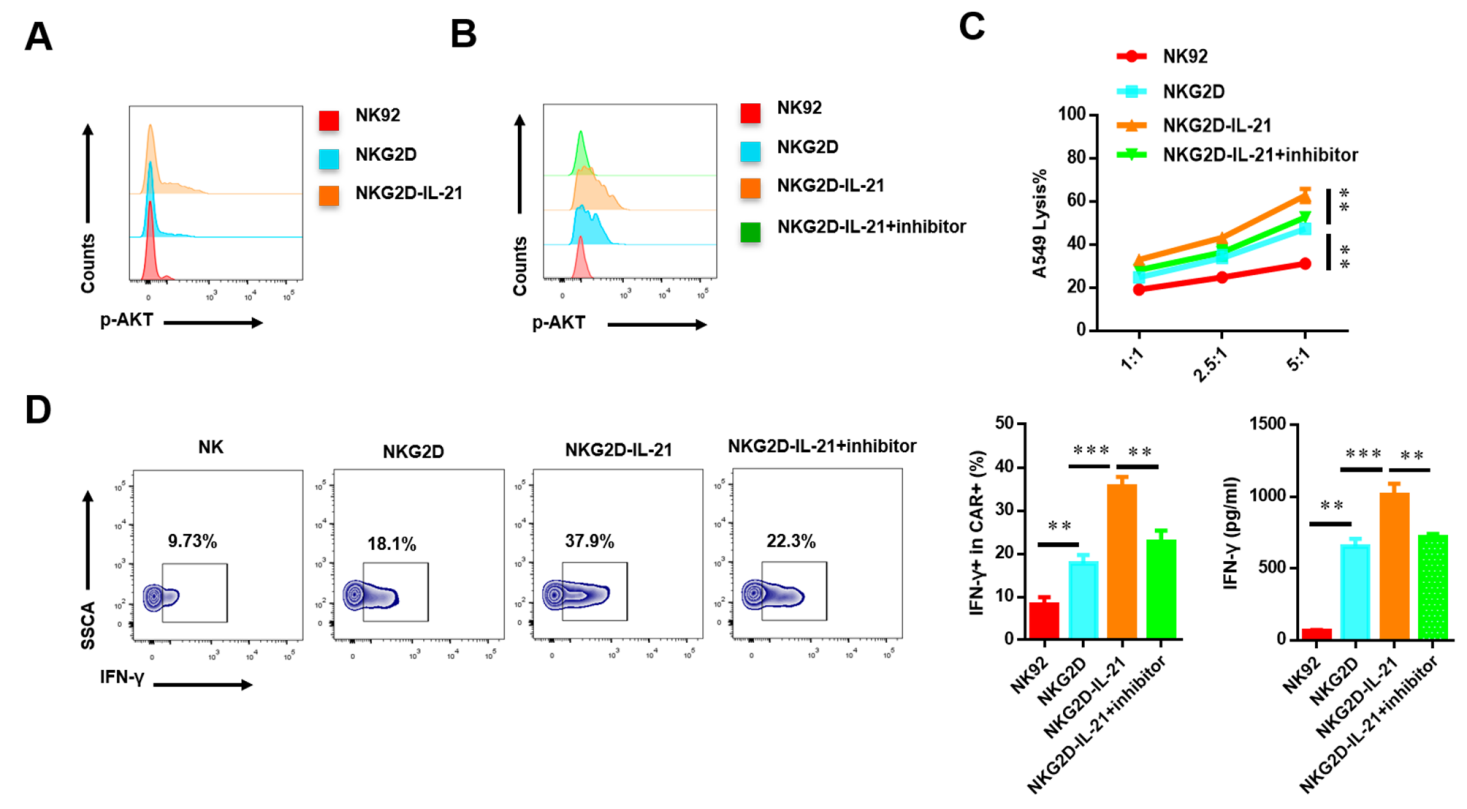
IL-21 regulates CAR-NK cells via AKT signaling pathway. (**A**) NK-92, NKG2D CAR-NK-92, and NKG2D-IL-21 CAR-NK-92 cells were co-incubated with A549 cells at a 5:1 ratio for 4 h. The expression of p-AKT was detected by flow cytometry. (**B**) NK-92, NKG2D CAR-NK-92, and NKG2D-IL-21 CAR-NK-92 cells were co-incubated with A549 cells and inhibitor of p-AKT at a 5:1 ratio for 4 h. The expression of p-AKT was detected by flow cytometry. (**C**) Line plots displaying the cytotoxicity of NK-92, NKG2D CAR-NK-92, and NKG2D-IL-21 CAR-NK-92 cells which were co-incubated with A549 cells at a different effector to target (E:T) ratios for 4 h in the absence and presence of the 20 µM LY294002 (PI3K inhibitor). (**D**) NK-92, NKG2D CAR-NK-92, and NKG2D-IL-21 CAR-NK-92 cells were co-incubated with A549 cells at 5:1 ratio for 4 h in the absence and presence of 20 µM LY294002 (PI3K inhibitor). The production of IFN-γ was detected by flow cytometry. Data were representative of three independent experiments. ** *P* < 0.01; *** *P* < 0.001

The PI3K/Akt signaling pathway has also been confirmed to participate in promoting IL-21-mediated T cell function in vitro. To explore whether IL-21 regulates the anti-tumor activity of CAR-NK-92 cells via the PI3K/Akt signaling pathway, we accessed the phosphorylation level of AKT. Our results showed that the expression of p-AKT was up-regulated (Fig. 5A). To further confirm whether IL-21 regulates the function of CAR- NK-92 cells through PI3K/Akt signaling pathway, the inhibitor of p-AKT was added. The result suggested that inhibitor significantly reduce the phosphorylation level of AKT, cytotoxic functions, and the production of IFN-γ (Fig. 5B-D). Therefore, IL-21 mediates the function of CAR-NK92 cells by PI3K/Akt signaling pathway.

### NKG2D-IL-21 CAR-NK-92 cells showed effective and persistent antitumor activity in vivo

**Fig. 6:**
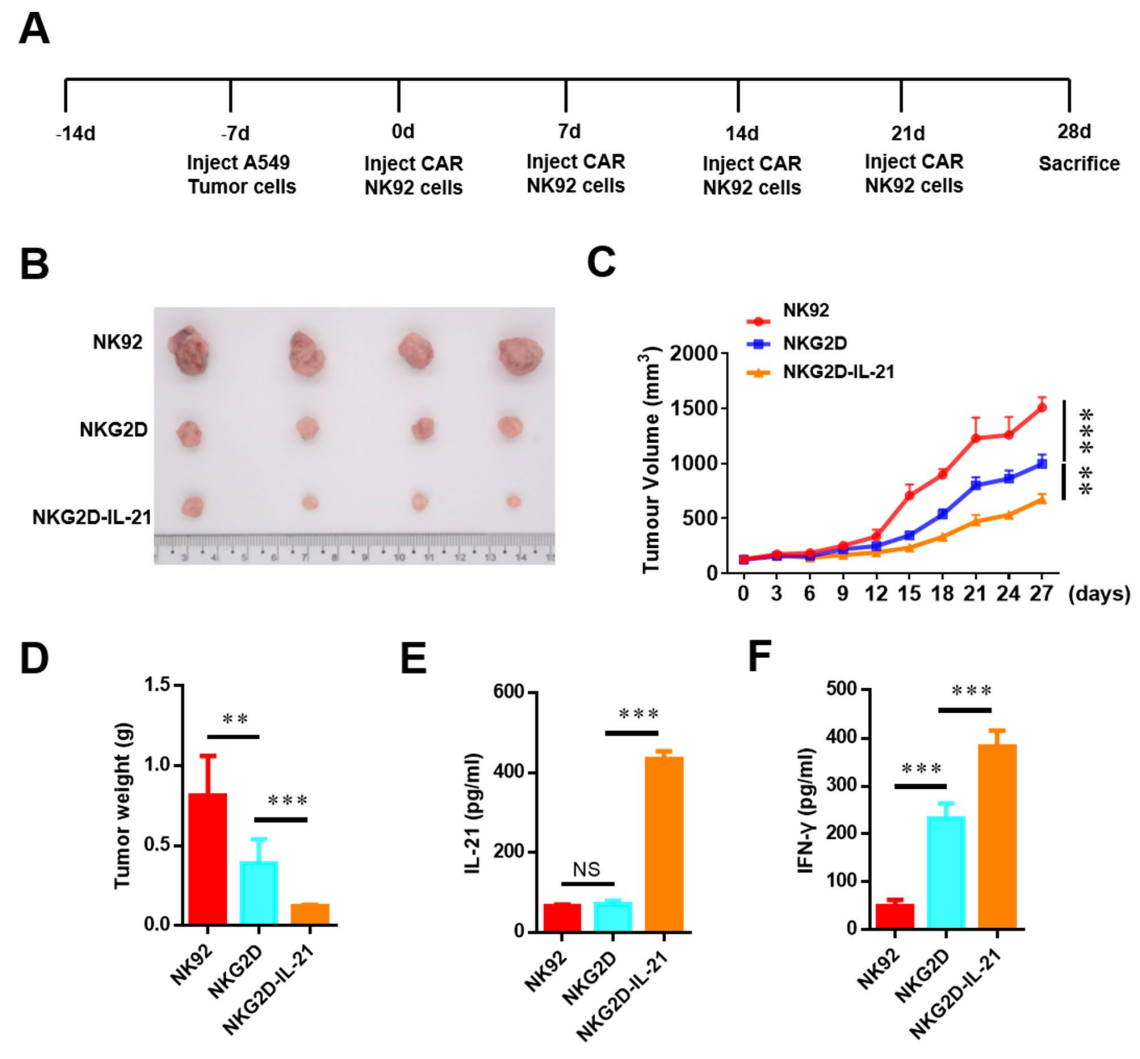
IL-21 showed effective anti-tumor activity in xenograft models. (**A**) Schematic diagram of the complete animal experiment (*n* = 5). (**B-C**) Tumor images of mice injected with A549 cells at the indicated time points. (**D**) Histogram of the weights of tumors. (**E-F**) The level of IL-21 and IFN-γ in serum measured by ELISA. Data were representative of three independent experiments. ** *P* < 0.01; *** *P* < 0.001

To further verify the anti-tumor efficacy of NKG2D-IL-21 CAR-NK-92 cells in vivo, we established subcutaneous xenografts by injecting A549 cells on the right flank of NSG mice. 7 days later, mice were administered with Mock-NK-92 cells, NKG2D CAR-NK-92 cells, or NKG2D-IL-21 CAR-NK-92 cells by intravenous injection (Fig. 6A). The tumor volumes in mice treated with NKG2D-IL-21 CAR-NK-92 cells were significantly lower than those treated with NKG2D CAR-NK-92 cells (Fig. 6B-D). Additionally, the NKG2D-IL-21 CAR-NK-92 cells group released a higher serum level of IL-21 and IFN-γ (Fig. 6E, F).

**Fig. 7:**
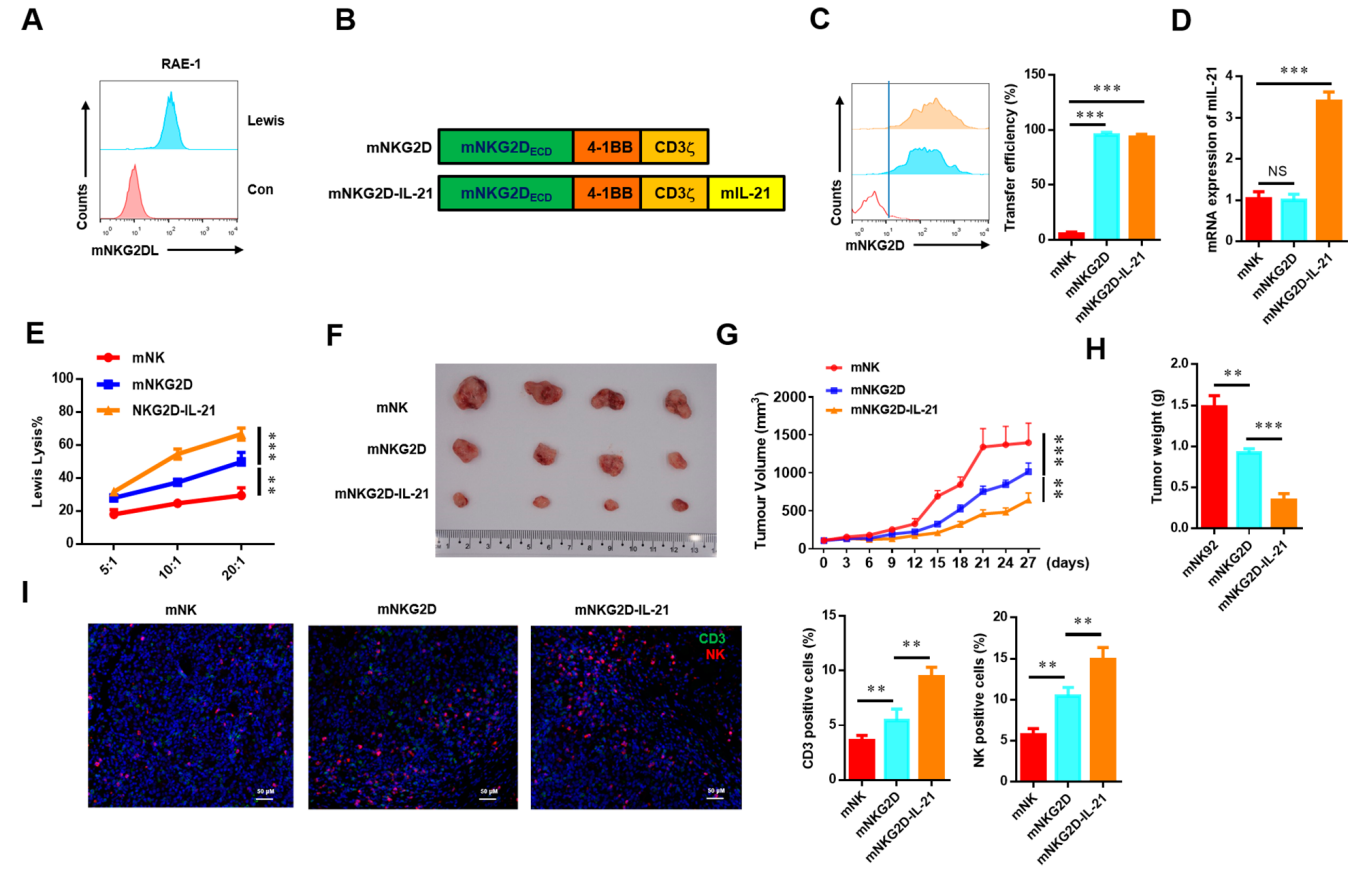
mIL-21 enhanced the anti-tumor activity of mNKG2D CAR-NK cells. (**A**) Schematic representation of mNKG2D (*n* = 5). (**B**) Flow cytometry analysis of the transfection efficiencies. (**C**) qRT-PCR analysis of IL-21 mRNA level in NK, mNKG2D CAR-NK, and mNKG2D-IL-21 CAR-NK cells. (**D**) Rae-1 expression of lung cancer cell line was detected by flow cytometry. (**E**) Line plots of the cytotoxicities of mNK, mNKG2D CAR-NK, and mNKG2D-IL-21 CAR-NK cells against the lung cancer cell line at a different effector to target (E: T) ratios for 4 h. (**F-G**) Tumor images of mice injected with A549 cells at the indicated time points. (**H**) Histogram of the weights of tumors. (**I**) Immunofluorescent staining revealed the expression of CD3 and NK1.1. Cell nuclei are stained with DAPI. Data were representative of three independent experiments. ** *P* < 0.01; *** *P* < 0.001

Next, we established the murine NKG2D-IL-21 CAR-NK cells to identify the previous results (Fig. 7A-D). mNKG2D-IL-21 CAR-NK cells displayed significantly higher specific cytolytic activity compared to mNKG2D CAR-NK cells against Lewis cells in vitro (Fig. 7E). Meanwhile, mNKG2D-IL-21 CAR-NK cells could obviously inhibit the tumor growth and enhance the infiltration of T cells and NK cells in vivo (Fig. 7F-I).

## Discussion

ACT has become a novel approach for treating cancer such as CAR-T cell therapy. However, the GvHD and cytokine storm are the main obstacles for the usage of CAR-T cells. Preclinical studies have demonstrated that CAR-NK cells exhibit in vivo activity comparable to that of CAR-T cells in xenograft murine models. Notably, the CAR-NK groups exhibit lower levels of cytokine release, suggesting a potentially more controlled and balanced immune response [47, 48].. NK cells can overcome these limitations because NK cells do not induce GvHD and release small amounts of cytokines [49, 50]. In addition, NK cells can be obtained from more allogeneic sources than T cells, for example, allogeneic donors or NK-derived cell lines. NK-92 is one of the NK cell lines which has shown better efficiency and safety in allogeneic cell therapy due to the decreased expressions of KIR and activating receptors [51, 52]. In this study, we biologically engineered NK cells to express NKG2D and IL-21 and explored the anti-tumor activities of these CAR-NK cells.

At present, numerous targets have been explored, such as EGFR, Her2, PSMA, CD19, and CD33 et al. NKG2D, a vital activating receptor, is one of targets and encoded by a Killer Cell Lectin Like Receptor K1 (KLRK1) and expresses on NK cells. NKG2D could bind to many ligands, including MICA, MICB and ULBP1-6 [53, 54]. Previous studies have found that NKG2DLs expressed on many tumors which were identified by NKG2D and activate NK cells and T cells [55, 56]. In our study, we compared the expressions of NKG2DLs on A549, H1975, and PC9 cell lines. We found that A549 and H1975 cells expressed higher levels of NKG2DLsthan PC9 cells. These results indicated that A549 and H1975 cells are the potential target lung cancer cells of our CAR-NK cells.

To enhance the function of CAR-NK cells, various modifications have been developed to address specific challenges. These modifications aim to improve the persistence of CAR-NK cells, enhance their tumor-targeting activity, prevent antigen escape by tumor cells, and provide better control over CAR expression. Some of these modifications include incorporating cytokines such as IL-2, IL-15, and IL-18 to support CAR-NK cell survival, activation, and cytotoxicity. Additionally, strategies involving the IL-2 receptor β chain (IL-2Rβ) coupled with STAT3/5 signaling have been explored to improve CAR-NK cell persistence and anti-tumor efficacy. Moreover, the inclusion of chemokine receptors like CCR2 can enhance the migration and infiltration of CAR-NK cells into tumor tissues, thereby improving their tumor-killing potential [53].. In our study, to enhance the anti-tumor activity of CAR-NK cells, we modified the CAR-NK cells expressing IL-21, which has been reported to increase the anti-tumor immunity in NK cells. As expected, the expression of IL-21 resulted in enhanced cytotoxic functions of NKG2D CAR-NK-92 cells against A549 and H1975 cells, expression of CD107a, and production of IFN-γ. Moreover, IL-21 could promote the proliferation of NKG2D CAR-NK-92 cells and reduce autologous apoptosis and exhaustion, which are beneficial for NKG2D CAR-NK-92 cells to resist tumors. To further verify the effect of NKG2D-IL-21 CAR-NK-92 cells, we established xenograft models by inoculating tumor cells subcutaneously in the right flank of the NOD/SCID mice. The PI3K/Akt and related pathways play a crucial role in internalizing the effects of external growth factors and membrane tyrosine kinases. Studies have reported that IL-21 can mediate multiple biological processes by activating the PI3K/Akt pathways, including responses to viral infections and inflammation. In line with these previous findings, our data also showed that NKG2D-IL-21 CAR-NK-92 cells significantly reduced tumor growth and promoted higher levels of IFN-γ compared with NKG2D CAR-NK-92 cells. In addition, our finding suggested that IL-21 mediated the anti-tumor activity of NKG2D CAR-NK-92 cells through the AKT signaling pathway.

In conclusion, our study demonstrated that IL-21 could enhance the anti-tumor activity of NKG2D CAR-NK cells and increase the release of INF-γ via AKT signaling pathway. Overall, our study would provide a novel approach for treatment of solid malignancies in clinics.

### Electronic supplementary material

Below is the link to the electronic supplementary material.

Supplementary Material 1

## Data Availability

The original contributions presented in the study are included in the article. Further inquiries can be directed to the corresponding authors.
