# Supplementary material for: Co-expression of IL-21-Enhanced NKG2D CAR-NK cell therapy for lung cancer
Source: BMC Cancer. 2024 Jan 23;24:119. doi: 10.1186/s12885-023-11806-1 (PMC10807083; doi:10.1186/s12885-023-11806-1)
Supplement: Supplementary file 1 — Supplementary Material 1 [file 12885_2023_11806_MOESM1_ESM.docx]

**A**


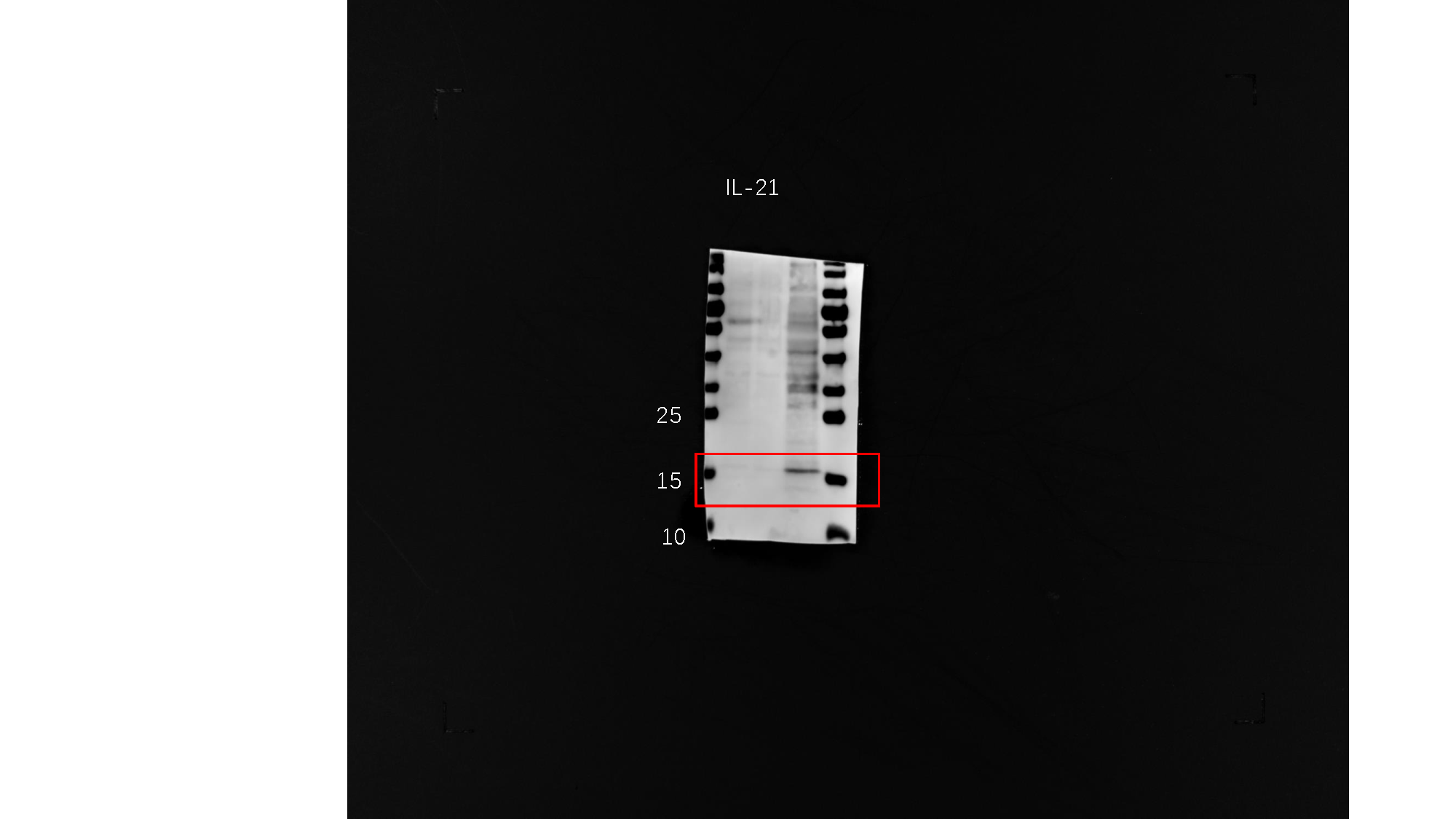


**B**


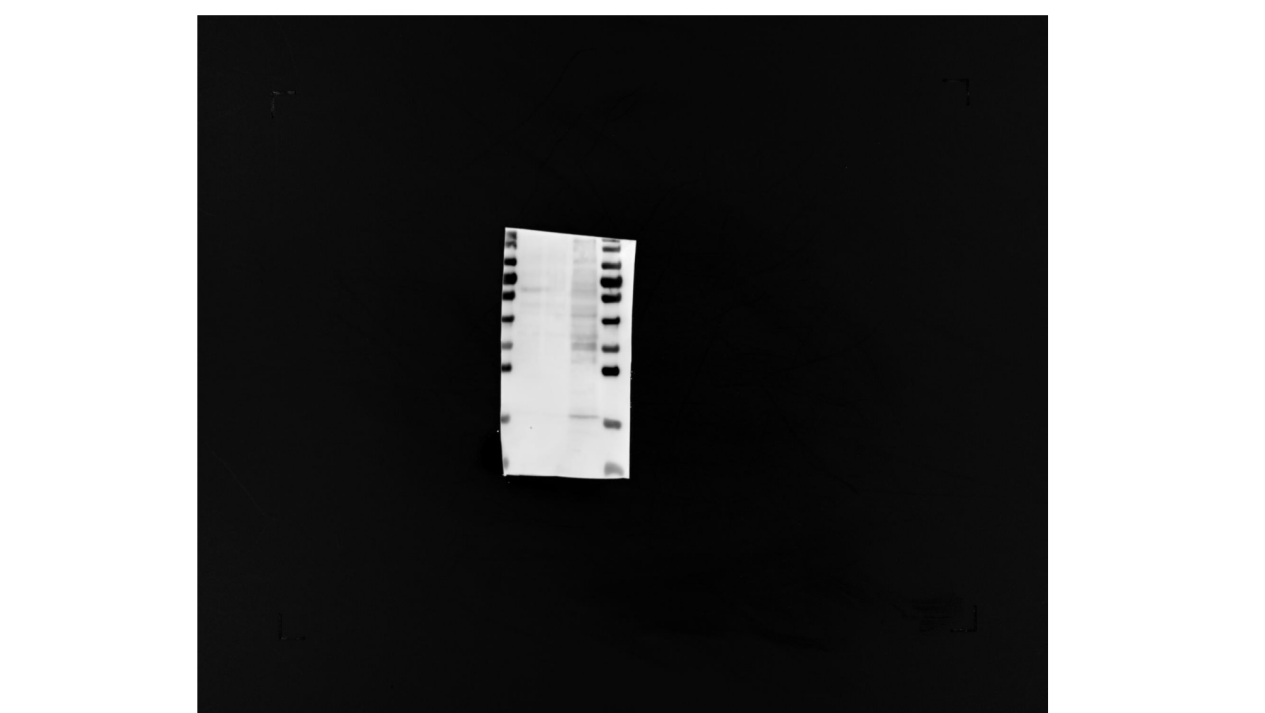


**C**


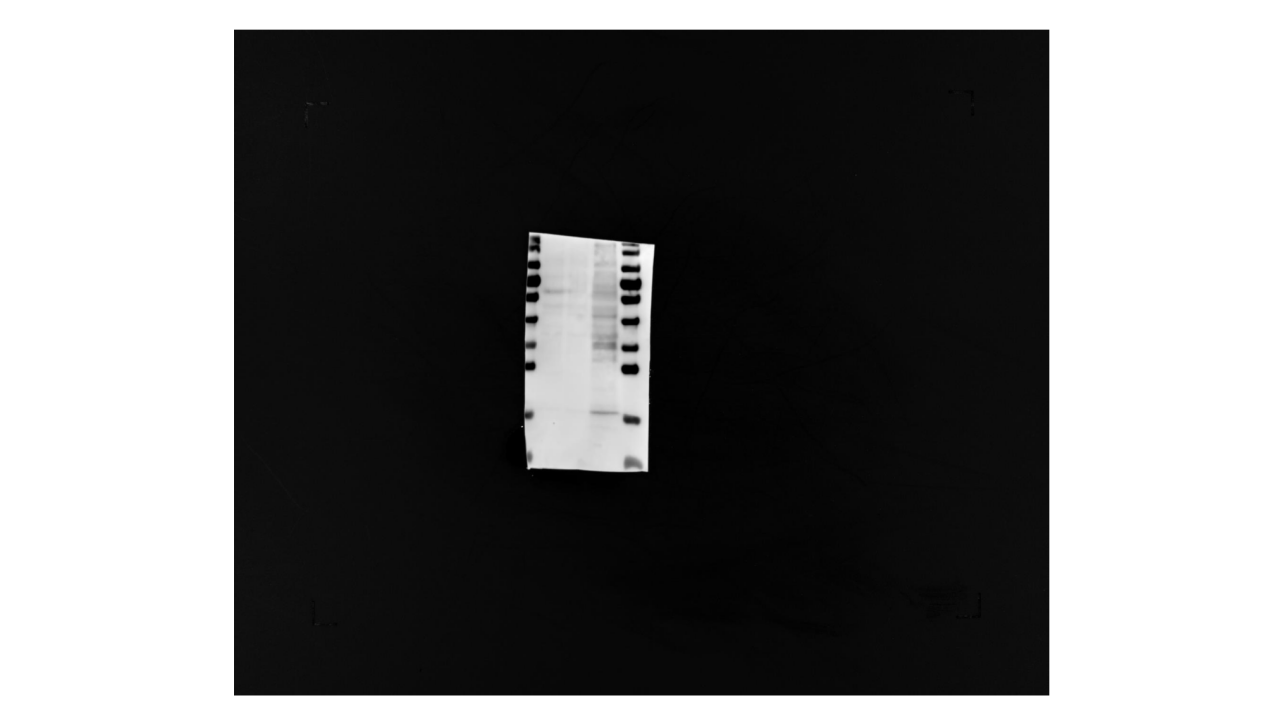


**D**


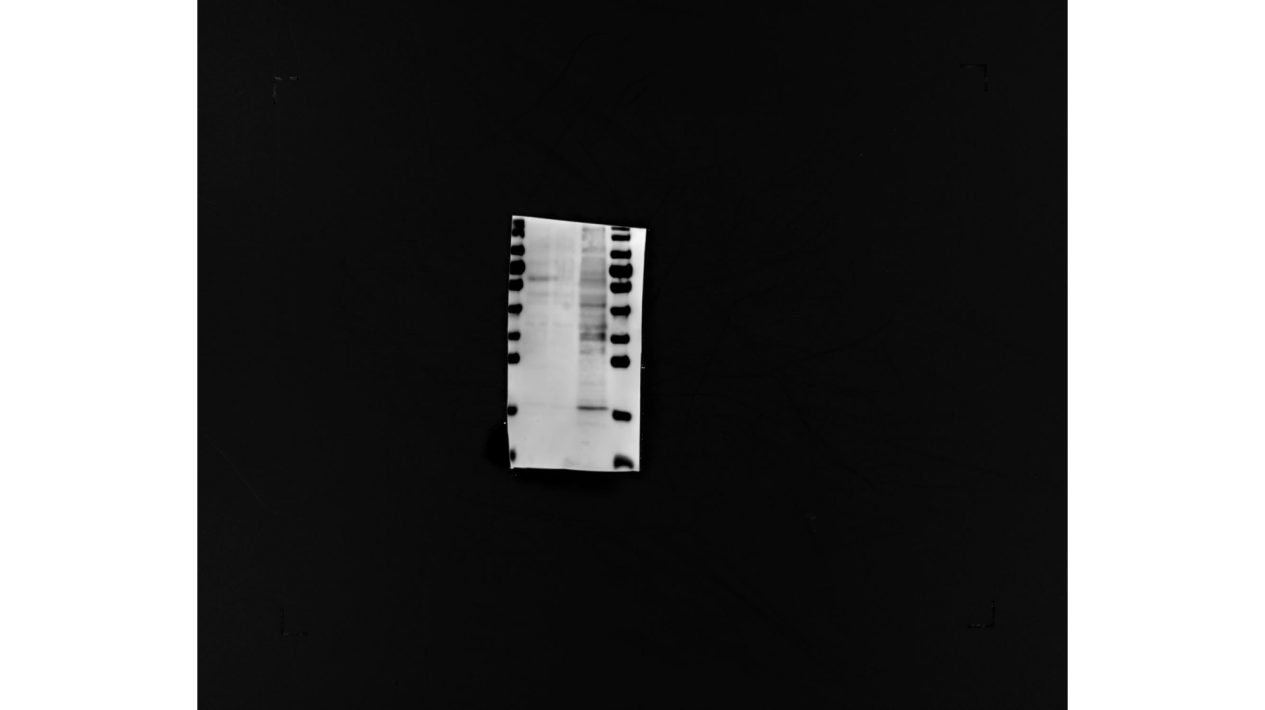


**E**


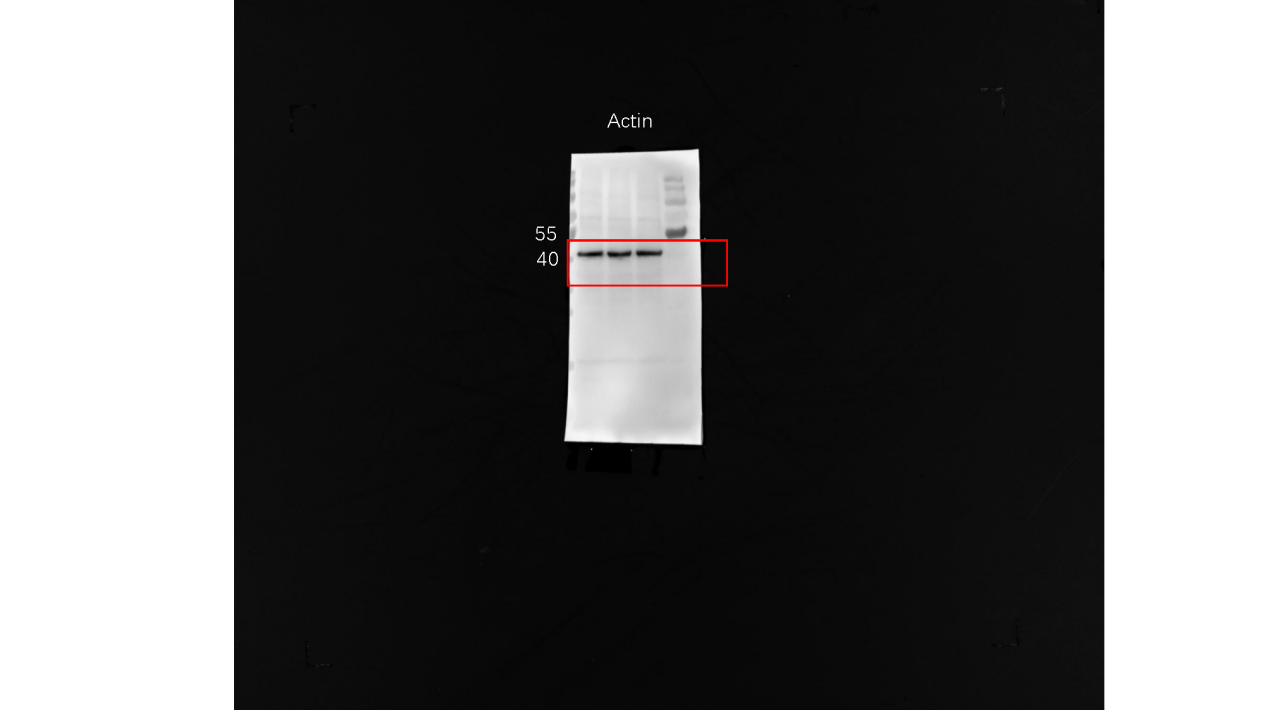


**F**


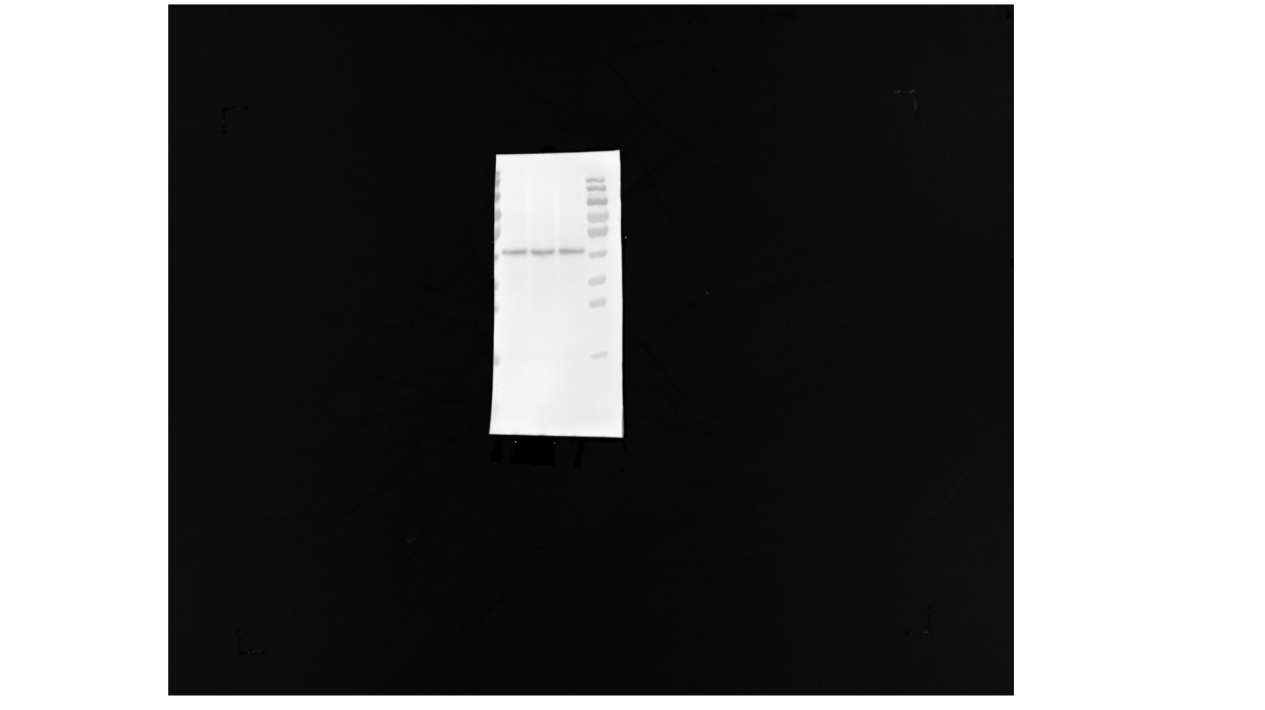


**G**


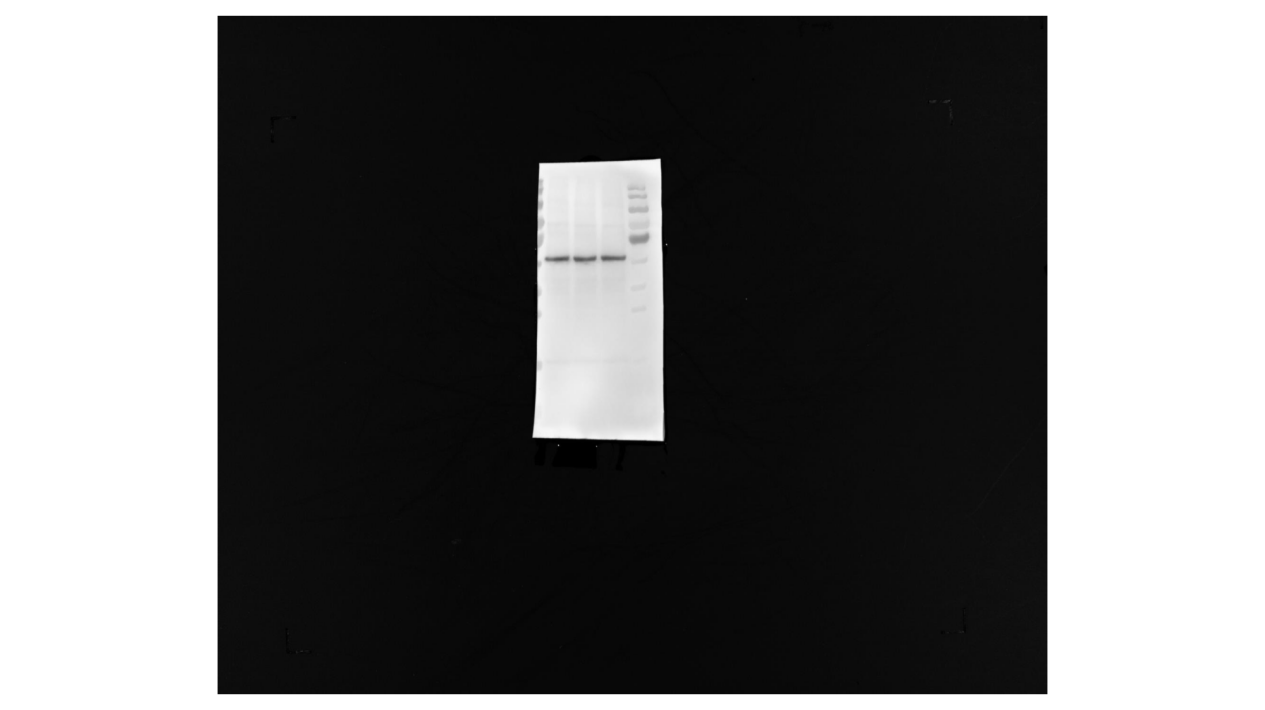


**H**


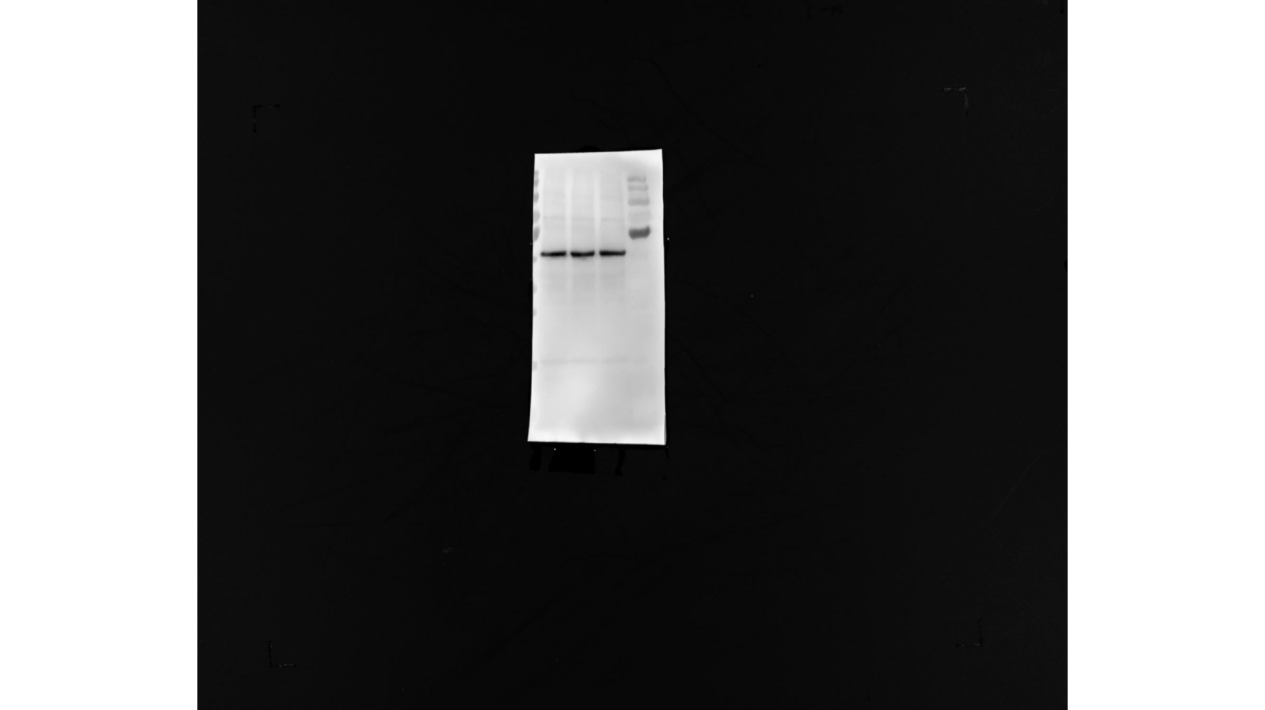


**SUPPLEMENTARY FIGURE 1. The unprocessed gels and blots with molecular size markings for Figure 2D.** The regions of the original blots used in **Figure 2D** are highlighted by red boxes. **A.** The images of IL-21 exposure for 20s. **B.** The images of IL-21 exposure for 5s. **C.** The images of IL-21 exposure for 10s. **D.** The images of IL-21 exposure for 15s. **E.** The images of Actin exposure for 5s. **F.** The images of Actin exposure for 2s. **G.** The images of Actin exposure for 3s. **H.** The images of Actin exposure for 4s.
